# Fibroblast-Like Synoviocytes Glucose Metabolism as a Therapeutic Target in Rheumatoid Arthritis

**DOI:** 10.3389/fimmu.2019.01743

**Published:** 2019-08-02

**Authors:** Patricia Gnieslaw de Oliveira, Mirian Farinon, Elsa Sanchez-Lopez, Shigeki Miyamoto, Monica Guma

**Affiliations:** ^1^Department of Medicine, School of Medicine, University of California, San Diego, La Jolla, CA, United States; ^2^Pharmacology, School of Medicine, University of California, San Diego, La Jolla, CA, United States

**Keywords:** rheumatoid arthritis, fibroblast-like synoviocytes, hexokinase-2, glucose metabolism, glycolytic inhibitors

## Abstract

Metabolomic studies show that rheumatoid arthritis (RA) is associated with metabolic disruption that may be therapeutically targetable. Among them, glucose metabolism and glycolytic intermediaries seem to have an important role in fibroblast-like synoviocytes (FLS) phenotype and might contribute to early stage disease pathogenesis. RA FLS are transformed from quiescent to aggressive and metabolically active cells and several works have shown that glucose metabolism is increased in activated FLS. Glycolytic inhibitors reduce not only FLS aggressive phenotype *in vitro* but also decrease bone and cartilage damage in several murine models of arthritis. Essential glycolytic enzymes, including hexokinase 2 (HK2) and 6-phosphofructo-2-kinase/fructose-2,6-biphosphatase (PFKFB) enzymes, have important roles in FLS behavior. Of interest, HK2 is an inducible enzyme present only in the inflamed rheumatic tissues compared to osteoarthritis synovium. It is a contributor to glucose metabolism that could be selectively targeted without compromising systemic homeostasis as a novel approach for combination therapy independent of systemic immunosuppression. More information about metabolic targets that do not compromise global glucose metabolism in normal cells is needed.

Rheumatoid arthritis (RA) pathogenesis includes synovial hyperplasia or *pannus*, which consists of accumulation of macrophages and fibroblast like synoviocytes (FLS) (1–4), resulting in enhanced invasiveness and destruction of adjacent cartilage and bone (3, 5). FLS are the major component of rheumatoid *pannus* and have a key role in its formation (4). In healthy individuals, these cells ensure the structural integrity of a normally organized synovial lining (6) and secretes hyaluronic acid and lubricin, two important constituents of synovial fluid that are responsible for lubricating the joint (7, 8). However, after acquiring an aggressive phenotype, FLS have reduced contact inhibition, resistance to apoptosis, increased migration, and increased ability to invade periarticular tissues including bone and cartilage (4, 9). These activated cells produce several mediators that induce angiogenesis, cell growth, and recruitment and activation of immune cells (4). In addition to contributing to the inflammatory environment, FLS also produce matrix metalloproteases (MMPs) that degrade the extracellular matrix and contributes to cartilage destruction (10).

Recently, an increasing number of studies have shown that FLS activation and the subsequent joint damage are associated with an altered metabolism which may be therapeutically targetable. The metabolism of all four major classes of macromolecules (carbohydrates, proteins, lipids, and nucleic acids) will change after cell activation. Carbohydrate metabolism is a fundamental biochemical process that ensures a constant supply of energy to living cells. The most important carbohydrate is glucose, which is first transported into the cell through glucose transporter 1 (GLUT1), then broken down via glycolysis by sequential metabolic enzymes [including hexokinase (HK), aldolase, phosphoglycerate kinase (PGK1), and pyruvate kinase] to generate pyruvate, and afterwards will either enter into the tricarboxylic acid (TCA) cycle and oxidative phosphorylation to generate ATP, or will be converted to lactate via lactate dehydrogenase (LDH).

Activation of FLS by hypoxia, platelet-derived growth factor (PDGF), tumor necrosis factor (TNF), and other inflammatory mediators increases glucose metabolism and transforms the FLS from quiescent to aggressive and metabolically active cells. Specifically, prior work—ours and others—has shown that glucose metabolism is increased in activated FLS, and glycolytic inhibition reduces not only FLS aggressive phenotype *in vitro* but also decreases bone and cartilage damage in several murine models of arthritis [(11–13); Table 1]. These works have suggested potential metabolic targets to reprogram metabolic disruptions and complement current therapies (11–13). In this brief review, we will summarize what is known about glucose metabolism in FLS and about potential metabolic therapies for RA that could modulate the aggressive behavior of FLS.

**Table 1:** Glycolyitic intermediate metabolites and their effect on RA FLS and animal models of arthritis.

**Intermediate**	**Enzyme**	**Trigger response**	**References**
Glucose		Glucose deprivation decreased IL-6, MMP-1 and MPP-3 production and the rate of proliferation and migration of FLS. In the SKG mouse model of arthritis, the glucose analog 2-DG decreased clinical score and thickness	(14, 15)
Fructose 1,6-bisphosphate	FBP1	Treatment with fructose 1,6-biphosphate reduced MPO activity, IL-6 and TNF-α joint levels, nociception, and neutrophil migration to the joint of mice with ZIA and AIA	(16)
Fructose 2,6-bisphosphate	PFKFB3	Inhibition of fructose 2,6-bisphosphate production decreased IL-6 secretion and proliferation, migration and invasion of FLS	(17, 18)
Glucose-6-phosphate	HK2	HK2 ablation decreased FLS invasive phenotype and also attenuated the severity of bone and cartilage damage in a mouse model of inflammatory arthritis	(19–21)
1,3-bisphosphoglycerate/ 3-phosphoglycerate	PGK1	Silencing of PGK1 decreased the secretion of IL-1β and IFN-γ as well as proliferation of FLS	
Pyruvate		Use of BrPa (a halogenated analog of pyruvate that inhibits glycolysis) decreased histologic score and levels of arthritis in K/BxN mouse models of arthritis	(14, 15, 20, 22)
Lactate	LDH/MCT	Increased levels of lactate induced FLS invasiveness	(17)
Succinate	SDH	Succinate induced fibrosis and angiogenesis and SDH inhibition attenuated the severity of rat CIA	(23, 24)

*FLS, fibroblast-like synoviocyte; FBP1, fructose 1,6-biphosphatase; 2-DG, 2-deoxy-D-glucose; MPO, myeloperoxidase; ZIA, zymosan-induced arthritis; AIA, antigen-induced arthritis; PGK1, phosphoglycerate kinase 1; BrPa, bromopyruvate; MCT, monocarboxylate transporters*.

## Glucose Metabolism Activation in the RA FLS

Glucose metabolism seems to be especially enhanced in joints with arthritis and involved in RA pathology. The high consumption of glucose by the RA joints can be visualized by PET imaging with 18F-FDG, a probe that detects glycolytic tissues (25). Synovial FLS and macrophages were shown to contribute to FDG-PET accumulation in the RA synovial tissue (26). The synovial tissue of RA patients also presents an enhanced level of lactate compared to non-inflamed synovial tissue (27). The local lower glucose levels and higher ratio of lactate to glucose in the RA synovial tissue suggest an increase in anaerobic cellular metabolism of resident cells, triggered by the inflammation and the hypoxic environment commonly detected in the RA joints (28, 29). This dysregulation is further suggested by the increase of lactate and glucose in the serum of RA patients (30). Moreover, glucose levels are lower in the synovial fluid of RA patients in comparison to non-inflamed synovial fluid (31). This is also supported by metabolic studies using mass spectrometry, which show differential metabolite profile in RA FLS and osteoarthritis (OA) FLS (32). Glycolysis, pentose phosphate pathway (PPP), and amino acid metabolism were different in RA FLS compared to OA FLS (32). In addition, FLS increased its intracellular levels of glucose after TNF stimulation (33).

Shift from oxidative phosphorylation to glycolytic ATP production is a common feature of activated and reactive cells like fibroblasts and macrophages. Micro environmental factors in the RA joint seem to potentiate this metabolic adaptation of FLS and macrophages. The synovial tissue is enriched in hypoxia-inducible factor 1 alpha (HIF1α), a transcription factor induced in hypoxic environments that contributes to RA pathogenesis at multiple steps (29), including supporting enhanced glycolytic activity. Among the HIF1α-transcriptionally regulated glucose metabolism related genes, GLUT1, HK2, and LDH are upregulated in RA FLS (14, 19, 34–36). The effect of HIF1α on glycolysis contributes to FLS survival (37), myeloid recruitment by FLS, angiogenesis (38), and FLS migration and invasion (29). In addition, it promotes the expression in RA FLS of inflammatory mediators that perpetuates interactions with other synovial cells including T and B cells (39, 40).

Other signaling pathways critical for FLS expression of adhesion molecules, pro-inflammatory cytokines, and MMPs, as well as for apoptosis inhibition, and for FLS migration and invasion are mitogen-activated protein kinases (MAPK), nuclear factor kappa B (NF-κB), and phosphoinositide-3-kinase (PI3K)/AKT (41–50). These pathways are activated by both hypoxia and inflammation. They also regulate glucose metabolism through several mechanisms including the upregulation of GLUT1 (51). These pathways are also involved in the phosphorylation of rate-limiting glycolytic enzymes, including 6-phosphofructo-2-kinase/fructose-2,6-bisphosphatases (PFKFB) and HKs (52, 53). JAK/STAT signaling, which also plays a role in FLS activation (54–56), was also shown to mediate glucose uptake and HK2 expression (57). Therefore, the phenotypic changes from FLS at rest to an activated and invasive state are coupled with metabolic alterations like increased GLUT1 and HK2 expression and lactate production (14, 17).

Likewise, the inhibition of glycolysis decreases the aggressive behavior of these cells by decreasing cytokine production, proliferation, migration, and invasion (14, 17). Three targetable glycolytic enzymes were recently shown to be involved in FLS aggressive phenotype. One is the bifunctional PFKFB3 enzyme, which converts fructose-6-phosphate to fructose-2,6-bisP (F2,6BP). F2,6BP is an allosteric activator of 6-phosphofructokinase-1 (PFK-1) and stimulates glycolysis overriding the inhibitory effect of ATP on PFK-1 (58). It was identified as a regulator of insulin/IGF-1 signaling pathway. Suppression of PFKFB3 was found to decrease insulin-stimulated glucose uptake, GLUT4 translocation, Akt signaling, and glycolytic flux (59). In FLS, PFKFB3 inhibition reduced glucose uptake which resulted in decreased lactate production (17, 18). The inhibition of the glycolytic flux by small molecule inhibitors of PFKFB3 significantly reduced FLS migration and invasion, and the production of inflammatory mediators (17). Of interest, FPFK15, a PFKFB3 inhibitor, not only suppressed glucose uptake and lactate secretion but also NF-κB and MAPK activation in RA FLS (18). The second enzyme is the rate-limiting enzyme HK2. Overexpression of HK2 in FLS provides a migratory and invasive advantage that is abolished when HK2 is ablated (19, 20). The last enzyme is phosphoglycerate kinase (PGK)1. Anti-PGK1 siRNA treatment of RA FLS not only decreased cell proliferation and cell migration, but also interleukin (IL)-1β and interferon (IFN)-γ secretion (21).

Since metabolic pathways are highly interconnected, other metabolic pathways described in activated FLS might also affect global glucose metabolism. Glutamine metabolism is increased in FLS, with glutaminase 1 (GLS1) playing a role in regulating the proliferation of these cells. FLS proliferation is reduced under glutamine-deprived conditions, or after GLS1 silencing or inhibition (60). Choline metabolism is also highly activated in FLS. Inhibition of choline kinase (ChoKα) suppressed the RA FLS aggressive phenotype by increasing apoptosis and decreasing cell migration (61). Glycogen synthase 1 (GYS1)-mediated glycogen accumulation was shown to block AMPK activation and to contribute to FLS phenotype as well (62). Finally, RA FLS also overexpresses the neutral amino acid transporter LAT1, and has an increased uptake of leucine after IL-17 stimulation, which potentiate FLS migratory capacity that was eliminated by blocking LAT1 (63). Other amino acids including tryptophan might also play a role in FLS phenotype (64).

## FLS Glucose Metabolism and Chronic Activation in RA

**Figure 1:**
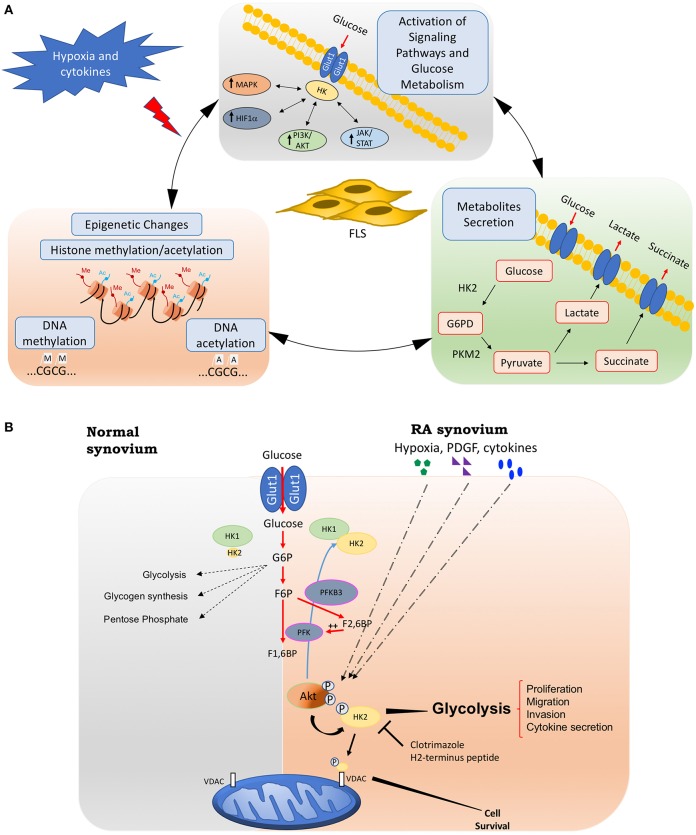
Fibroblasts-like synoviocytes (FLS) glucose metabolism and chronic activation in RA. **(A)** Chronic glucose metabolic changes induced by hypoxia and inflammatory mediators in FLS will activate many signaling pathways, including HIF, MAPK, PI3K/Akt, and JAK/STAT pathways, which also increases the expression of key glucose metabolism related genes such as GLUT1, HK2, or LDH. Intermediate glucose metabolites including pyruvate, lactate, succinate, a-ketoglutarate, fumarate, and acetyl-coenzyme will create a chronic and sustained FLS activation, either by being secreted extracellularly and triggering profound effects on the biology of other cells, or by inducing a new epigenetic landscape that results in a stable FLS activation that is maintained even without continuous stimulation. **(B)** Hypoxia, growth factors, and cytokines in arthritis synovium stimulate Akt phosphorylation, which will up-regulate HK2 expression and HK2 phosphorylation. The phosphorylation of HK2 by Akt is accompanied by an increased binding of the enzyme to mitochondrial outer membrane voltage-dependent anion channel (VDAC). Binding to VDAC enhances the affinity of hexokinases. Therefore, HK2 mitochondrial binding might promote glucose metabolism and FLS invasive phenotype. Mitochondrial HK2 might also inhibit apoptosis. Thus, mitochondrial association of HK2 might promote resistance to growth, invasion, and apoptosis of RA FLS, which contribute to joint destruction in RA. Selective HK2 mitochondrial dissociation might be an attractive potential selective target for arthritis therapy and safer than global glycolysis inhibition. HK2, hexokinase 2; G6PD, glucose 6 phosphate dehydrogenase; PKM2, pyruvate kinase muscle isozyme M2; PFK, phosphofructokinase; PFKB3, 6-phosphofructo-2-kinase/fructose-2,6-biphosphatase 3; G6P, glucose-6-phosphate; F6P, fructose-6-phosphate; F1,6BP, Fructose 1,6-bisphosphate; F2,6FB, Fructose 2,6-bisphosphate; VDAC: voltage-dependent anion channel.

Although researchers have suggested a role for metabolic alterations in RA pathology, we are far from understanding which changes are normal responses to cell activation and are transient metabolic responses to acute inflammation, and which are the result of damage and chronic activation that could play a role in driving the pathology of RA. These chronic metabolic changes in FLS can have not only profound effects on the biology of other cells through intermediate metabolites but also can create a new epigenetic landscape that results in a stable FLS activation that is maintained even without continuous stimulation (Figure 1A).

Some pro-inflammatory mediators increase several TCA cycle intermediates, and emerging evidence show that these intermediates classically associated with metabolic functions also possess signaling functions as inflammatory mediators and drive chronic activation. For instance, metabolic profiling has revealed itaconic acid as a potential marker of RA, and TNF increased its concentration in the K4IM human fibroblast cell line (65). Importantly, the increased levels in itaconic acid can be attenuated by treatment with infliximab, a biologic drug targeting TNF (65). Succinate is another TCA cycle intermediate that is abundant in RA synovial fluids. Synovial succinate correlates with enhanced release of IL-1β by macrophages in a mechanism that involves the overexpression of succinate receptor Sucnr1/GPR91 (66). In addition, Sucnr1/GPR91 functions as a chemotactic signal for recruitment of dendritic cells into lymph nodes which leads to Th17 cells expansion in a murine model of inflammatory arthritis (67). In FLS, succinate has a myofibroblast effect on FLS (23). A recent paper also evaluated Sucnr1/GPR91 in FLS and concluded that both intra- and extracellular succinate play a role in synovial angiogenesis. Intracellular succinate induced angiogenesis through HIF1α induction, while extracellular succinate increased vascular endothelial growth factor (VEGF) through GPR91 receptor (24).

Pyruvate is another metabolite generated during glycolysis and is converted to acetyl-CoA to fuel the TCA cycle. Acetyl-CoA is an important cofactor that catalyzes the transfer of an acetyl group. Histone acetyltransferases (HATs) are enzymes that use this co-factor and regulate histone acetylation and therefore link metabolism and epigenetics in cells (68, 69). Other metabolites elevated after glucose metabolism activation such as fumarate, succinate, and lactate also modify chromatin and nucleic acid-modifying enzymes activity by competitively inhibiting substrate utilization (68). The relationship between metabolic intermediates and chromatin-modifying enzymes implies that metabolic changes could directly affect gene expression by modulating chromatin-modifying enzymes and triggering epigenetic dysfunction (68, 69). Of interest, several reports have shown that epigenetic alterations, such as histone modification, might contribute to RA pathogenesis (70). In fact, a comprehensive epigenomic characterization of RA FLS has recently been described (71), suggesting that synovial fibroblasts stimulation results in a stable activation that is maintained even without continuous stimulation through epigenetic changes. Further FLS studies are needed to better understand the epigenetic modifications affecting metabolic gene expression and glucose metabolism that can drive chronic RA FLS activation and may help to identify novel metabolic targets.

## Glucose Metabolism Targets in RA FLS

The concept of metabolic reprogramming to improve immunotherapy and to complement current therapies is being slowly translated into the autoimmune disease field (11, 72–74). In fact, glycolytic inhibitors not only reduce FLS aggressive phenotype *in vitro* but also decrease bone and cartilage damage in several murine models of arthritis. More specifically, ablation of glycolytic genes or treatment with 3-bromopyruvate, which antagonizes HK2, significantly reduced the severity of several murine arthritis models (14, 15, 19, 20, 22). Although HK2 specific inhibitors are not available, HK2 can be inhibited by the use of 2-deoxyglucose (2-DG), which is a derivative of glucose that can be phosphorylated by HK2 but not mobilized through succeeding steps of glycolysis. Murine studies have shown that 2-DG reduces cell proliferation and the severity of a spontaneous model of RA arthritis (15). HK2 ablation has also attenuated the severity of bone and cartilage damage in a murine model of inflammatory arthritis (19). Interestingly, the administration of fructose 1,6-bisphosphate (FBP), a glycolytic intermediate, decreased arthritis scores in two different animal models. Mechanistic studies showed that this metabolic intermediate activated the anti-inflammatory adenosinergic pathway instead of enhancing FLS glycolysis (16). Treatment with a saponin that inhibits succinate dehydrogenase (SDH) activity ameliorated the clinical symptoms of the arthritis as well as histopathologic features of synovial hyperplasia, infiltration of inflammatory cells, and fibrosis (23). In addition, treatment with dimethylmalonate, another inhibitor of SDH, decreased succinate content in the synovial tissue of rats with collagen-induced arthritis (CIA) in addition to amelioration of the disease (24). Finally, inhibition of the enzyme ChoKα (61) and GLS1 (62) also ameliorated the severity of experimental autoimmune arthritis.

Yet, although all these works have demonstrated a role of glucose metabolism in RA, inhibiting global glucose metabolism is not desirable. In addition, inhibition of some of the above pathways can have other detrimental effects. For instance, a recent report demonstrated a key anti-inflammatory function of HIF1α by driving the expression of IL-10 in B cells (75). PFKFB3 activity is also defective in CD4 T cells in RA patients which results in energy deprivation that prone cells to undergo apoptosis (76). Thus, there is a need of finding specific metabolic targets that are induced in activated FLS.

Out of all the glycolytic enzymes described to play a role in RA pathogenesis, HK2 could function as a selective metabolic target (Figure 1B). HKs catalyze the phosphorylation of glucose to glucose-6-phosphate (G6P) that facilitates glucose entry into cells. G6P initiates several metabolic pathways that need glucose, including glycolysis, the hexosamine pathway, glycogen synthesis, and the PPP (77). HK2 also plays important roles in angiogenesis (78). HKs has four different isoforms: HK1 is the ubiquitous isoform in all adult issues. However, HK2 is an inducible isoform that is only highly expressed in skeletal and cardiac muscles, and adipose tissue (77). HK2 is also highly upregulated in tumor cells and HK2 inhibition synergies with anti-tumor treatment and improves response to therapy (79). In addition to its canonical metabolic roles in tumor or cardiac tissues, HK2 translocates to the nucleus or mitochondria and triggers an autophagic and anti-apoptotic responses through its interaction with the voltage-dependent anion channel (VDAC) (80, 81). Of interest, HK2 plays a small role in inflammation driven by T cells, so HK2 inhibition should have limited immunosuppressive effects (82). Importantly, we and others have shown that the synovial expression of HK2 is elevated only in RA compared to OA samples (19, 20). Given HK2 selective overexpression in inflamed RA synovium, its small role in T cells, and its expression in a very limited number of adult tissues, HK2 is an attractive selective target for arthritis therapy that is safer than global glucose metabolism inhibition (19). In addition to its expression profile, its diverse effects at various cellular compartments could offer another level of specificity since targeting a specific intracellular compartment of HK2 (i.e., cytosol, nucleus, or mitochondria) would also provide a selective means to block deleterious effects of this enzyme in RA without affecting glucose metabolism in normal cells. Therefore, HK2 could be selectively targeted offering a safer and novel additional approach for combination therapy in RA joint disease independent of systemic immunosuppression.

Of interest, rheumatologists already have antimetabolites in the current RA armamentarium, such as methotrexate and leflunomide. Although they were thought to inhibit the proliferation of synovial and immune cells, methotrexate and other disease-modifying antirheumatic drugs (DMARDs) also have effect on glucose metabolism. For instance, methotrexate treatment significantly reduced HK2 expression and glucose/fructose carriers (SLC2A5, a member of the solute carrier family 2) in human FLS, suggesting that FLS glycolytic activity can be modulated by methotrexate (83). Anti-TNF treatment decreases the synovial expression of GLUT1 and of the glycolytic enzymes pyruvate kinase muscle isozyme M2 (PKM2) and glyceraldehyde 3-phosphate dehydrogenase (GAPDH) in patients that responded to TNF inhibition compared to non-responders (17). Anti-IL-6 receptor therapy inhibited oxidative stress and improved endothelial function in RA leucocytes, although whether or not this therapy also has a metabolic effect on the synovial tissue is not known yet (84). Finally, inhibition of JAK/STAT3 signaling with tofacinib, a drug approved for severe RA and active psoriasis, induces oxidative phosphorylation and maximal respiratory capacity of FLS while shutting down key glycolytic enzymes including HK2 and LDH. This effect correlated with the reduction of inflammatory mediators and FLS activation (85).

## Conclusion

Growing evidence suggests that the study of activated metabolism not only of immune cells but also of stroma cells including FLS can provide critical pathways for therapeutic intervention. Pre-clinical studies in mouse models of inflammatory arthritis strongly suggest that agents that interfere with certain steps of glycolysis can be therapeutic in RA and have identified potential targetable glycolytic enzymes such as HK2, and glycolytic intermediate metabolites (Table 1). In addition, therapeutic effects of DMARDs could be due, at least partially, to the inhibition of glucose metabolism, highlighting the pathogenic role of this metabolic pathway. As global inhibition of glucose metabolism is not desirable, more information about inducible glycolytic genes, the specific distribution of these targets, their effect in different cellular compartments, and their additional non-metabolic functions, may help us to identify new targets that do not compromise global glucose metabolism in normal cells.

## Author Contributions

All authors listed have made a substantial, direct and intellectual contribution to the work, and approved it for publication.

### Conflict of Interest Statement

The authors declare that the research was conducted in the absence of any commercial or financial relationships that could be construed as a potential conflict of interest.
